# Supplementary material for: Circular RNA circIGF2BP3 Promotes the Proliferation and Differentiation of Chicken Primary Myoblasts
Source: Int J Mol Sci. 2023 Oct 24;24(21):15545. doi: 10.3390/ijms242115545 (PMC10650573; doi:10.3390/ijms242115545)
Supplement: Supplementary file 1 [file ijms-24-15545-s001.zip › ijms-2611812-supplementary.pdf]

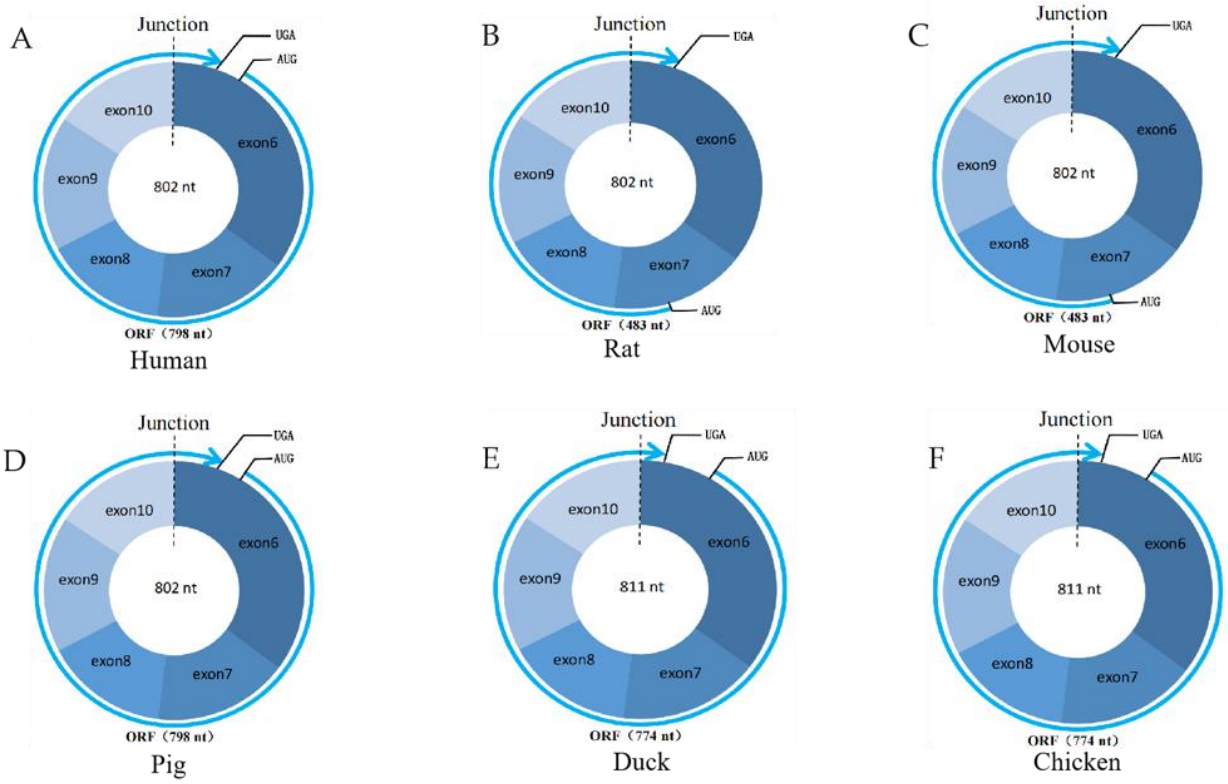

Figure S1. (**A**–**F**) Structures of circIGF2BP3 in different species.

Table S1. Sequence conservation of circIGF2BP3 in different species (%).

	**Human**	**Rat**	**Mouse**	**Pig**	**Duck**	**Chicken**
Human	100	90.65	90.40	91.27	78.91	77.81
Rat		100%	95.89	88.03	76.45	75.09
Mouse			100	88.90	77.44	77.07
Pig				100	77.93	78.30
Duck					100	94.08
Chicken						100
